# Arthroscopic repair of traumatic rotator cuff tear following total shoulder arthroplasty: A case report and review of literature

**DOI:** 10.1002/ccr3.7210

**Published:** 2023-05-04

**Authors:** Alvarho Guzman, Caleb Shin, Miles Gamboa, Didi Wu, Therese Dela Rueda, Sarah Jenkins, Patrick McGahan, James Chen

**Affiliations:** ^1^ Advanced Orthopaedics and Sports Medicine San Francisco California USA; ^2^ Albany Medical College Albany New York USA

**Keywords:** orthopedics, sports medicine

## Abstract

**Key Clinical Message:**

We highlight the rare case of arthroscopic repair of a traumatic tear following total shoulder arthroplasty. Moreover, there is no reported literature describing the arthroscopic repair of a rotator cuff tear after total shoulder arthroplasty.

**Abstract:**

This case report highlights an arthroscopic rotator cuff repair involving full‐thickness tears of the supraspinatus and infraspinatus after a total shoulder arthroplasty performed 7 years prior. To our best knowledge, no published literature exists highlighting the arthroscopic repair of a traumatic rotator cuff tear following total shoulder arthroplasty.

## Graphical abstract

AP view of operative shoulder displaying intact shoulder arthroplasty and humeral shaft hardware following arthroscopic rotator cuff repair.

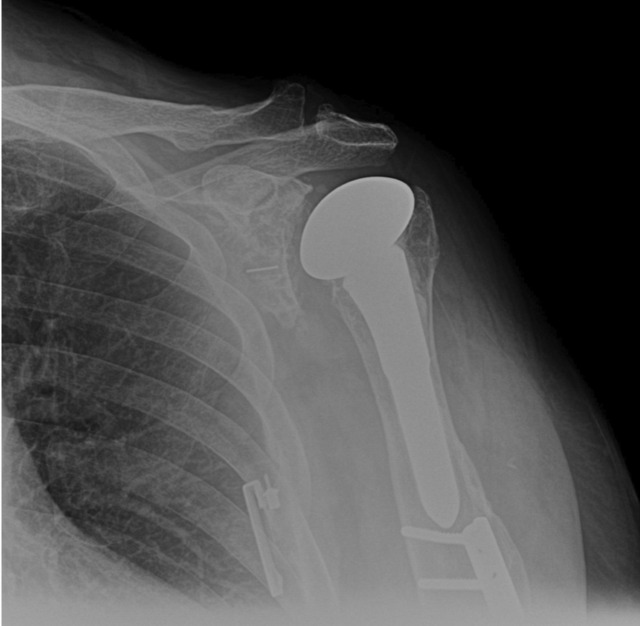

## 1 INTRODUCTION

Total shoulder arthroplasty (TSA) is one of the most common and successful surgeries in orthopedics to treat a variety of shoulder conditions including primary osteoarthritis, rheumatoid arthritis, avascular necrosis, and severe fractures involving the glenohumeral joint.^1^, ^2^ Over the past two decades, the number of TSA cases performed each year within the United States has risen exponentially due to better prosthetics, more advanced surgical techniques, and research substantiating its clinical effectiveness in decreasing shoulder pain and restoring shoulder function.^3^, ^4^ As expected, accompanying this significant rise in TSA cases is an overall rise in the number of revision surgeries and postoperative complications, the most common of which are infection, instability, component loosening, neurologic defects, and rotator cuff tearing of both traumatic and non‐traumatic nature.^3^, ^4^, ^5^, ^6^ This case study analyzes the clinical success of an arthroscopic rotator cuff repair (RCR) performed in a patient with a pre‐existing TSA to further evaluate the effectiveness of this procedure in treating post‐TSA rotator cuff complications.

In this case report, we describe the arthroscopic RCR of a patient with an existing TSA performed 7 years prior. To our knowledge, there is no available literature highlighting the arthroscopic repair of a traumatic rotator cuff tear following a previous TSA.

## 2 CASE PRESENTATION

A 62‐year‐old right‐hand dominant male who received a left TSA 7 years prior presented to our clinic with persistent left shoulder pain after a fall he sustained 2 months ago. The patient also sustained a humerus fracture several years prior for which he subsequently received a humeral shaft ORIF. He presented with pain in the superior left shoulder extending distally to the biceps region in addition to weakness with forward flexion and overhead motions. On physical examination of the left shoulder, he demonstrated a painful active and passive range of motion, decreased strength with abduction and external rotation, and a positive Neer's test and empty can test. CT arthrogram imaging of the left shoulder was reviewed which the patient had brought to his appointment. Imaging depicted an uncomplicated left prosthesis from his previous arthroplasty and a full‐thickness tear of the anterior fibers of the infraspinatus extending into the posterior fibers of the supraspinatus, with a delaminating infraspinatus component.

The risks, benefits, and alternatives to nonoperative treatment and surgical treatment were thoroughly discussed with the patient and consisted of converting the left TSA to a left reverse TSA, left RCR, and conservative treatment of physical therapy, non‐steroidal anti‐inflammatory drugs, and rest. The patient was informed that converting from a left TSA to a reverse TSA would be a difficult operation. Successful RCR would also require that the rotator cuff tendon be anchored to healthy humeral bone amenable to healing. Other surgical concerns include wear and tear of the TSA glenoid component, as indicated by the CT report. Based on the current examination, CT arthrogram results, and the patient's desire to increase the function of the left shoulder, left RCR was recommended. The patient expressed understanding of the explanations and agreement with the proposed treatment plan and elected to proceed with surgery.

**FIGURE 1:**
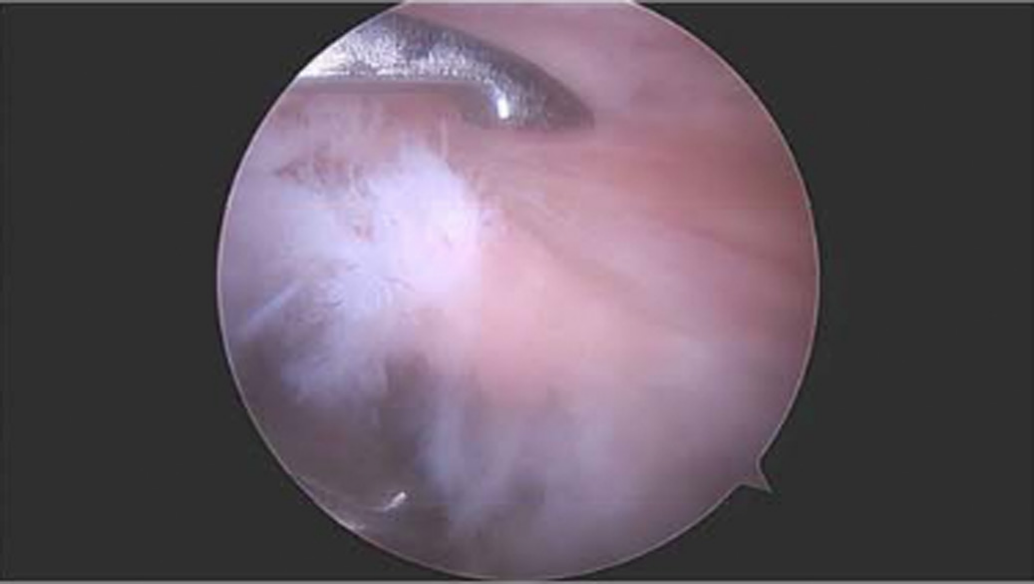
Patient positioned in the beach chair decubitus position. Arthroscopic image of the left shoulder through the anterior portal with a 30‐degree arthroscope demonstrating the torn supraspinatus with fraying.

The patient was placed in the beach chair position after administration of preoperative antibiotics, a peripheral nerve block, and general anesthesia. After the left upper extremity was prepped and draped in the usual sterile fashion and all bony prominences were padded, a surgical pen was used to mark bony anatomical landmarks. A #11 blade was used to create the posterior portal and the glenohumeral joint was entered with a blunt trocar and scope sheath. Standard diagnostic arthroscopy revealed intact glenoid and humeral head components of the TSA component and full‐thickness tears of the infraspinatus and supraspinatus with fraying (Figure 1). The anterior portal was created with a #11 blade, which was then dilated with a trocar and entered with a nonaggressive shaver to debride scar tissue. The arthroscope was then removed, the subacromial space was entered through the posterior portal with a blunt trocar and scope sheath, and a #11 blade created the lateral percutaneous portal with needle localization. Subacromial decompression was then performed and 6 mm of acromion was debrided with electrocautery from the bursal surface of the inferior border of the acromion.

**FIGURE 2:**
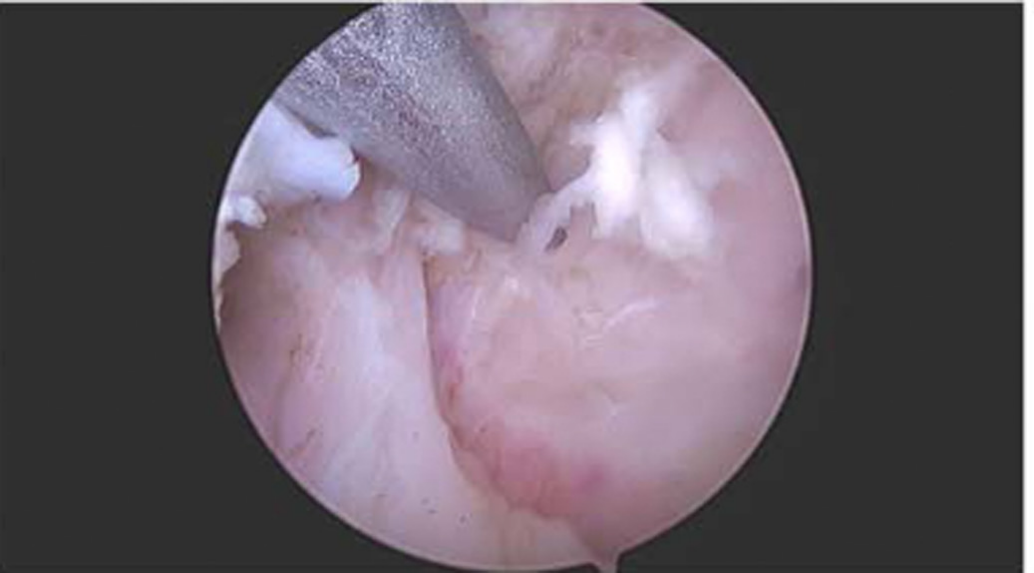
Patient positioned in the beach chair position. Arthroscopic image of the left shoulder through the anterior portal with a 30‐degree arthroscope depicts the humeral head prior to pilot hole creation. The pilot hole is created lateral to the articular margin of the humeral head anterior to the midline.

Rotator cuff repair was then performed using the SpeedBridge double‐row technique (Arthrex). Electrocautery prepared the periosteum of the humerus. Pilot holes were carefully positioned to avoid interference with TSA implants and created lateral to the articular margin of the humeral head anterior to the midline (Figure 2). A 4.75‐mm SwiveLock anchor (Arthrex) was then loaded and anchored securely into the pilot hole. This process was repeated, securing a second SwiveLock anchor (Arthrex) into the bone.

**FIGURE 3:**
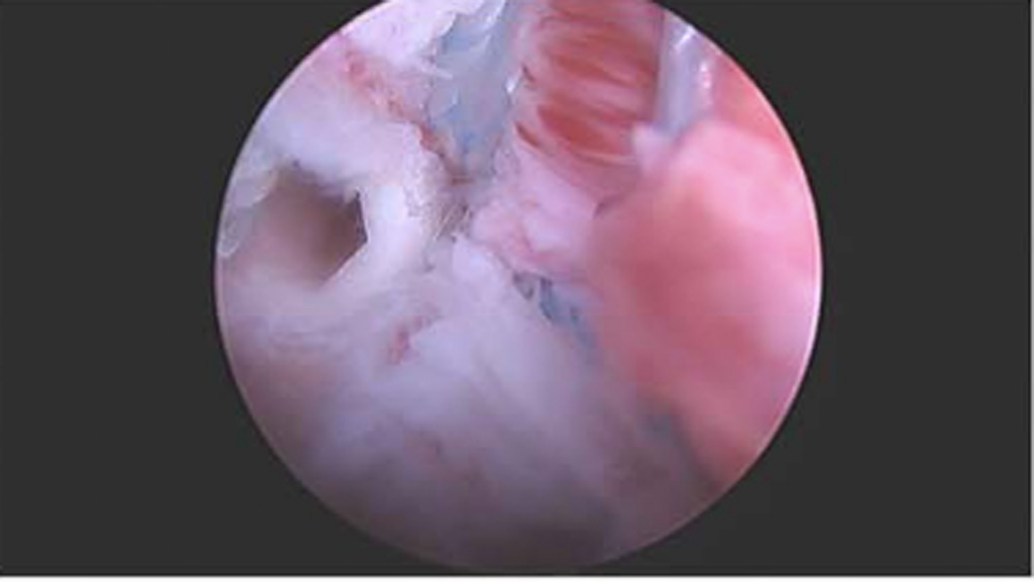
Patient positioned in the beach chair position. Arthroscopic image of the left shoulder through the anterior portal with a 30‐degree arthroscope depicts a completed rotator cuff repair following fixation with a second suture anchor, which revealed good lateralization of the supraspinatus at the footprint of the greater tuberosity.

The swaged tail of the suture loop was then loaded onto and passed through the Scorpion Suture passer (Arthrex) through the rotator cuff. The suture was then retrieved and the swage is cut to make two separate tails. This process was again repeated with the other loaded SwiveLock anchor (Arthrex). The suture tails were separated by cutting the ends with FiberWire (Arthrex) scissors, one tail from each anchor was pulled out through the lateral portal, and a punch created another pilot hole onto the greater tuberosity laterally. Two suture tails were then loaded onto a 4.75 mm SwiveLock anchor (Arthrex) and securely screwed into the pilot hole, which was repeated a second time with another set of suture tails and an additional SwiveLock anchor (Arthrex). Finally, a scope was inserted through the lateral portal, which revealed good lateralization of the supraspinatus at the footprint of the greater tuberosity and the RCR was complete. Intraoperative radiographs were taken to confirm that the implant was intact (Figure 3).

**FIGURE 4:**
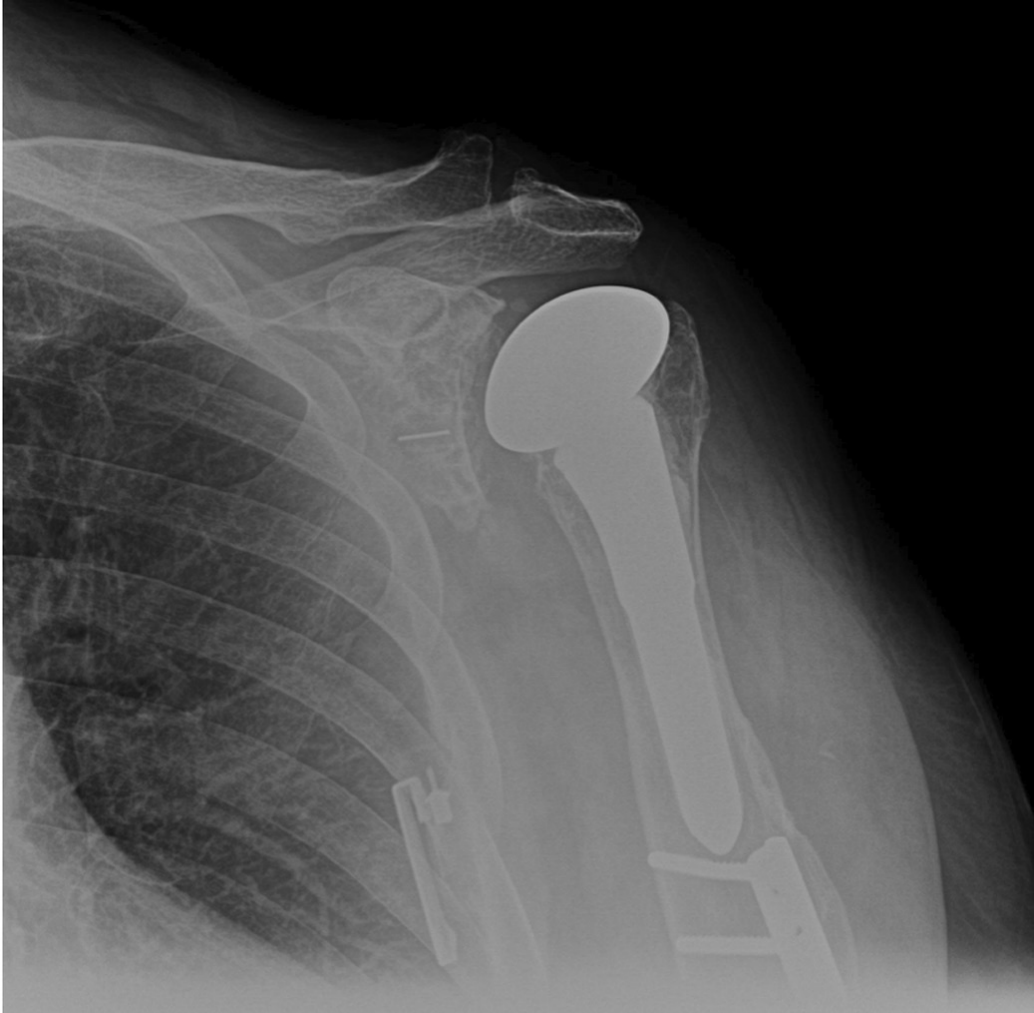
Postoperative radiographs of the left shoulder in anterior to posterior (AP) view depicting intact total shoulder arthroplasty hardware following arthroscopic rotator cuff repair. Intact humeral shaft ORIF without osseous complication is also seen.

Postoperatively, the patient was immobilized in a sling for 6 weeks. Dressing, bandages, and sutures were removed at the first postoperative visits where afterward formal physical therapy commenced. Postoperative radiographs revealed intact TSA implants (Figure 4). He is currently over 6 months out from surgery and is doing well with his rehabilitation. The patient reports good improvements in range of motion with associated mild anterior shoulder pain. In his most recent physical examination 6 months postoperatively, the patient demonstrated a full, painless active range of motion with forward flexion of 180°, external and internal rotation of 90°, and 5/5 strength of the supraspinatus, infraspinatus, and subscapularis. He has begun the strengthening portion of his physical therapy protocol and is expected to make a full recovery.

## 3 DISCUSSION

In patients who have already undergone TSA, rotator cuff tearing is a relatively uncommon yet detrimental post‐operative complication that may significantly impair overall shoulder function.^1^, ^2^, ^6^ Post‐TSA cuff tearing counteracts the functional improvements originally made by the TSA by decreasing shoulder range of motion, strength, and increasing pain.^1^, ^2^, ^5^, ^7^ In addition to these mechanical issues, post‐TSA cuff tearing may also compromise the stability of the TSA implants through proximal migration of the humeral component and loosening of the glenoid component.^1^, ^2^ Multiple studies have shown rotator cuff tears following TSA to have an incidence rate of about 2%–4%, although some studies such as the one published by Young et al. report even higher incidence rates of up to 16.8%.^1^, ^2^, ^8^ Although Blalock et al. used indirect radiologic evidence (x‐ray imaging) to diagnose rotator cuff dysfunction, a less accurate method than MRI or CT imaging, studies that use longer follow‐up periods after the TSA nonetheless report higher incidence rates of rotator cuff tearing due to age‐related degeneration of soft tissues in the shoulder and the impacts which the TSA implants have on shoulder biomechanics.^5^

Although rotator cuff dysfunction is relatively uncommon after TSA, even more rare are traumatic rotator cuff tears after TSA, with an estimated incidence of 1.3%–4% of cases.^1^ The prevalence of re‐operation to surgically address these tears is low with a re‐operation rate of about 1.2%.^1^ One solution to this problem that has become increasingly popular is the revision of the TSA to a reverse TSA (rTSA).^5^ Despite relatively high complication rates of revision rTSAs, shoulder function, and pain are successfully restored in most cases.^6^, ^9^ The less common alternative, a direct RCR, has been shown to be an effective treatment in certain situations, but more studies are necessary to better assess the clinical indications and outcomes for this option. Multiple factors such as the size of the cuff tear, location of the tear, quality of the torn tissue, and physical exam findings all contribute to determining whether the option for direct RCR is available.^5^

While the literature is well established with concomitant total shoulder replacement and RCR or rotator cuff dysfunction after TSA, our review of the literature found only three articles reporting RCRs after a TSA, with two out of the three reporting primarily on subscapularis insufficiency.^7^, ^8^, ^10^ The current literature disagrees in the management of these tears with some camps promoting non‐operative management, while other camps report success with surgical repair.^1^, ^7^, ^8^, ^10^ One study was by Miller et al.^7^ reported a case series of seven patients who experienced subscapularis tendon rupture after TSA repaired with an open deltopectoral approach. If the subscapularis was deemed irreparable, a pectoralis major tendon transfer was performed.^7^ The data were mixed in regards to the mechanism of injury with three patients reporting trauma, and four patients reporting no trauma.^7^ Moreover, while the results of range of motion postoperatively showed satisfactory results, patient outcomes in regard to pain and satisfaction varied.^7^ Another study by Hattrup et al.^8^ reported only 4 out of 18 cases in which patients with a rotator cuff tear after shoulder replacement healed satisfactorily. Although RCR commonly led to relief of pain, the range of motion was not improved or restored and thus they concluded that although successful RCR after shoulder replacement is possible, failure is more common.^8^

Secondary rotator cuff dysfunction following TSA is uncommon yet has been reported in orthopedic literature, with both superior glenohumeral subluxation and proximal humeral migration contributing to cuff dysfunction pathology after arthroplasty.^11^, ^12^, ^13^ A level IV evidence study by Young et al. analyzed secondary rotator cuff dysfunction following TSA for glenohumeral arthritis and found that at an average of 103 months postoperatively, the rate of secondary dysfunction was 16.8% and patients with secondary dysfunction had significantly worse clinical outcomes, radiographic loosening, and glenoid component migration.^2^ Bohsali et al.^14^ have also reported superior instability of the shoulder in 19% of TSA complications secondary to glenoid component loosening. Moreover, Khan et al.^15^ found one‐third of patients who received TSA for osteoarthritis developed superior migration of the humeral head 10 years postoperatively. Ikard et al.^16^ also cited that patients with high humeral heads were significantly associated with early postoperative dysfunction.

One of the difficulties, in this case, was ensuring that there was enough remaining bone in the greater tuberosity to insert the anchors. In patients with similar pathologies, we would recommend using the shortest anchors available to ensure that they can be fully inserted into the bone. One of the risks with this surgery is peri‐prosthetic fracture so it is important that your assistant use caution when punching the hole for the anchors. Intraoperative fluoroscopy should be used to ensure that implant is avoided. A full list of pearls and pitfalls is highlighted in Table 1.

**TABLE 1:** Pearls and pitfalls.

Pearls	Pitfalls
Ensure adequate space remains in greater tuberosity when establishing medial row‐suture anchors	Improper portal positioning can make placing anchors challenging especially given proximity of arthroplasty hardware to tear
Use shorter‐length suture anchors to ensure full insertion in bone given limited space within greater tuberosity	Risk of periprosthetic fracture to arthroplasty hardware from anchor insertion within bone
Caution when punching holes in greater tuberosity for anchor placement to avoid impaction of bone and arthroplasty hardware	Weak tensioning of sutures due to improper anchor placement

## 4 CONCLUSION

To our knowledge, this is the first case of a patient with isolated, full‐thickness tears of both the infraspinatus and supraspinatus following a TSA repaired with an arthroscopic approach. With this case report, we aim to highlight the successful arthroscopic RCR in a patient with an existing TSA 7 years prior in hopes of increasing research and awareness of this pathology for patients after shoulder replacement.

## AUTHOR CONTRIBUTIONS

**Alvarho J Guzman:** Conceptualization; data curation; formal analysis; investigation; methodology; project administration; resources; software; supervision; validation; visualization; writing – original draft; writing – review and editing. **Caleb Shin:** Conceptualization; data curation; formal analysis; investigation; methodology; project administration; resources; software; supervision; writing – original draft; writing – review and editing. **Miles Gamboa:** Data curation; investigation; methodology; resources; writing – original draft; writing – review and editing. **Didi Wu:** Conceptualization; investigation; methodology; project administration; resources; writing – original draft. **Therese Dela Rueda:** Conceptualization; methodology; project administration; resources; software; supervision; visualization; writing – review and editing. **Sarah Jenkins:** Project administration; resources; supervision; writing – review and editing. **Patrick McGahan:** Methodology; project administration; supervision; visualization; writing – review and editing. **James Chen:** Conceptualization; methodology; project administration; supervision; validation; writing – review and editing.

## FUNDING INFORMATION

None.

## CONFLICT OF INTEREST STATEMENT

J.L.C. is an educational consultant for Arthrex and receives compensation for medical educational lectures and instruction only.

## CONSENT

The authors obtained the patient's informed consent for print and electronic publication of the case report before submission.

## Data Availability

The data used to support the findings of this study are included in the article.
